# Advancing Gene Therapy for Phenylketonuria: From Precision Editing to Clinical Translation

**DOI:** 10.3390/ijms26178722

**Published:** 2025-09-07

**Authors:** Inseon Yu, Jaemin Jeong

**Affiliations:** Department of Biohealth Convergence, College of Science and Convergence Technology, Seoul Women’s University, Seoul 01797, Republic of Korea; dlstjs89@gmail.com

**Keywords:** Phenylketonuria (PKU), gene therapy, adeno-associated virus, animal model, base editing

## Abstract

Phenylketonuria (PKU) is an inherited disorder caused by mutations in the *phenylalanine hydroxylase (PAH)* gene that result in the amino acid phenylalanine (Phe) building up in the blood. Current therapies suggest low-*Phe* dietary management and (6R)-L-erythro-5,6,7,8-tetrahydrobiopterin (BH_4_) therapy, which are limited in efficacy and require lifelong treatment. Recent advances in gene therapy, including gene editing and viral-mediated gene delivery, produce therapeutic effects. Advancements in gene editing technologies, notably adenine base editors (ABEs) and CRISPR-based systems, in conjunction with enhanced delivery methods such as lipid nanoparticles (LNPs) and recombinant viruses, have demonstrated substantial promise in preclinical studies. This review details the pathophysiology of PKU treatment, and progress in preclinical and clinical gene therapy strategies. Emphasis is on adenine base editing using LNPs, recombinant adeno-associated virus (rAAV)-mediated gene transfer, and the translational challenges associated with these technologies. We also discuss future directions for therapeutic reach and ensuring long-term safety and efficacy.

## 1. Introduction

Phenylketonuria (PKU) is an autosomal recessive metabolic disorder with an estimated global incidence of approximately 1 in 10,000 to 15,000 children [1]. First described in 1934 by Dr. Ivar Følling [2], PKU is caused by a deficiency in *phenylalanine hydroxylase* (*PAH*), an enzyme primarily expressed in the liver that catalyzes the hydroxylation of *phenylalanine* (*Phe*) to *tyrosine* (*Tyr*). When *PAH* activity is deficient or absent, *Phe* accumulates to neurotoxic levels in the blood and brain, disrupting neurotransmitter synthesis and causing irreversible neurological damage if not treated early [3].

The standard of treatment for PKU includes strict dietary restriction of *Phe* intake starting in infancy, typically through medical foods and protein substitutes [4]. And there are several pharmacological options, such as sapropterin dihydrochloride (sapropterin; a synthetic form of (6R)-L-erythro-5,6,7,8-tetrahydrobiopterin (BH_4_), a *PAH* cofactor), pegvaliase (a pegylated bacterial enzyme that degrades *Phe*), and others, that provide alternatives for some patients [5]. Sapropterin enhances residual PAH activity and is effective only in a subset of patients with BH_4_-responsive genotypes, demonstrating a modest reduction in blood *Phe* levels in clinical trials (~29% over 6 weeks in responders) [6]. Pegvaliase lowers *Phe* levels independently of *PAH* via enzymatic degradation. Phase III trials demonstrated a ~62% reduction. However, it requires daily injections and carries a risk of immune-mediated adverse events, including anaphylaxis [6,7,8]. A third pharmacologic option, sepiapterin (PTC923), is currently under FDA review with a PDUFA date of 29 July 2025. In phase III studies, sepiapterin showed a mean reduction of 63% in plasma *Phe* levels and enabled over 97% of patients to liberalize their dietary protein intake. This makes sepiapterin a potentially more effective and better-tolerated oral alternative [6], but it is not universally effective and is associated with side effects.

A paradigm-shifting approach is gene therapy, which directly targets PKU mutations located in the PAH gene. Genome editing via CRISPR-Cas9 nucleases, adenine base editors (ABEs), and prime editors have only recently emerged as a potential tool for therapeutic interventions in inherited metabolic disorders. They are being delivered using both lipid nanoparticle (LNP) and recombinant adeno-associated virus (rAAV) platforms [9,10,11]. Delivery based on LNP systems allows for transient, non-integrating expression with lower immunogenicity and thus a potential for repeat dosing and reduced clinical safety concerns [12]. In contrast, rAAV vectors enable efficient hepatic delivery and long-term transgene expression, which is more suitable for diseases such as PKU, where sustained liver cell correction may be required [13]. For example, delivery of base editors in vivo using adeno-associated virus (AAV) has enabled consistent correction of a disease-relevant mutation (*PAH c.1222C>T* [*p.Arg408Trp*]) in humanized mice models of PKU, resulting in metabolic recovery and corrected blood Phe levels [14].

While several reviews have described PKU strategic management in the rare disease landscape, few of these actually articulate precision editing platforms together with putative pre-clinical animal models and early clinical trials emerging from this new field. This review intends to fill this gap by creating a complete synthesis through the transition from traditional drugs to the newest genome manipulation techniques (i.e., genome editing), emphasizing their translational potentials and proposing new perspectives. The current review will provide readers with an overview of the topic, differentiating from the status of research on PKU.

## 2. Pathophysiology and Genetic Basis of PKUs

### 2.1. PAH Gene Location and Enzyme Function

The *PAH* gene is located on 12q23.2 and encodes the hepatic enzyme PAH with the cofactor tetrahydrobiopterin (BH_4_) [15,16]. This reaction is an essential step in amino acid metabolism and is required for catecholamine, dopamine, and norepinephrine synthesis. But due to mutations in the *PAH* gene, this enzymatic conversion is impaired, resulting in the accumulation of toxic *Phe* in the blood. More than 3300 different mutations in the *PAH* gene have been reported, affecting enzyme function, stability, and expression [17]. These comprise missense, nonsense, splice-site, and frame-shift mutations. Hillert et al. carried out an analysis of PAH gene variants found in over 16,900 PKU individuals from 51 countries and demonstrated that the frequency distribution varied among regions [18]. The most common in Europe was *p.Arg408Trp* (*R408W*), largely present in Eastern and Central European populations. In Latin America, *p.Val388Met* (*V388M*), also known as *c.1163G>A*, was the most commonly observed variant [allele frequency (AF) 13.9%], while in East Asia, the *c.728G>A* (*p.Arg243Gln; R243Q;* AF = 21.8%) was predominant [18]. This illustrates the importance of these various genetic backgrounds, as the efficacy of PKU therapy is frequently determined by the phenotype, which can directly correlate with at least certain types of *PAH* mutation. BH_4_ therapy, used to supplement residual *PAH* activity, comprises one of the main treatment options now available for PKU. Nevertheless, the success rate of BH_4_ treatment is very much related to the type of *PAH* mutation, as well as different region-based frequencies. This approach does not have broad applicability to the treatment of many variants of *PAH*, as it relies mainly on individual patients having some residual *PAH* enzyme activity that can be stimulated by BH_4_ [19]. Therefore, BH_4_ cannot be used as a universal therapy for all individuals with PKU. As a result, BH_4_ cannot be an effective therapy for all individuals with PKU. This highlights the need for genotype-linked personalized treatment strategies.

### 2.2. Clinical Manifestations

For healthy individuals, blood *Phe* concentrations generally fall within the range of 50 to 110 μmol/L. The clinical classification of hyperphenylalaninemia and PKU is usually based on the level of untreated *Phe*. When *Phe* levels are between 120 and 600 μmol/L, the condition is referred to as mild hyperphenylalaninemia. Mild PKU is defined by levels ranging from 600 to 900 μmol/L. Concentrations between 900 and 1200 μmol/L indicate moderate PKU. Classic PKU is diagnosed when *Phe* concentrations exceed 1200 μmol/L before treatment initiation [20]. In untreated individuals, *Phe* levels usually exceed 1200 μmol/L, resulting in a spectrum of neurological and psychiatric complications [21]. High *Phe* concentrations cause competitive transport across the blood–brain barrier with large neutral amino acids, limiting uptake of *Tyr* and *tryptophan*, appropriate dopamine and serotonin precursors in the brain, and provoking an apoptotic phenomenon that takes place later in life [22]. It manifests with developmental delay, seizures, eczema, and autism-like behaviors related to the abnormal synthesis of melanin that leads to hypopigmentation. Even with early treatment, PKU patients as a whole show executive function deficits and attentional problems, as well as anxiety common in adults [23]. The lack of treatment in these highlights the importance of early therapeutic interventions aimed at normalizing *Phe* metabolism in children, and the reports of adherence and treatment as time progresses are very concerning [24]. Prenatal testing exists for PKU through chorionic villus sampling or amniocentesis. Also, non-invasive prenatal testing has been studied as a possible early option for screening in high-risk couples [25].

### 2.3. Limitations of Current Therapies

**Figure 1:**
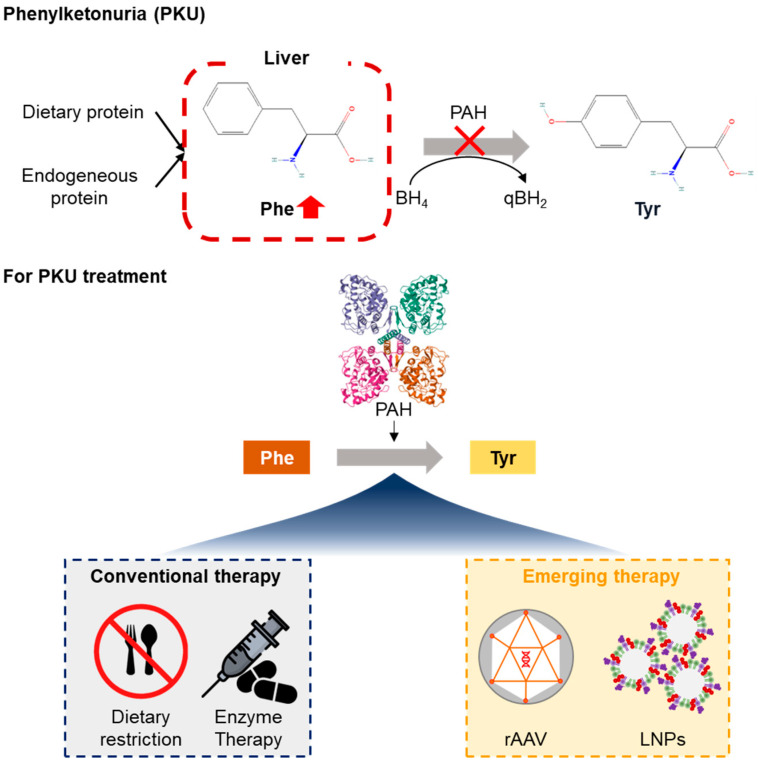
The pathological mechanism of PKU and therapeutic strategies. Conventional therapies include dietary restriction and enzyme therapy (sapropterin, oral drug; pegvaliase, subcutaneous injection). Emerging therapies include recombinant adeno-associated virus (rAAV)-mediated gene transfer and lipid nanoparticle (LNP)-delivered base editing.

The dietary restriction of *Phe* continues to be the mainstay of PKU management (Figure 1, Table 1). The only treatment is a lifelong low-*Phe* diet, supported by medical foods and amino acid formulas. It is difficult to subject oneself to a medical diet; however, the prescribed foods often have unpleasant tastes and impose restrictions on the social lives of both children and parents, compounded by dietary monotony. The risk of neurocognitive deficits is high due to poor compliance, especially in the adolescent and adult periods [26,27]. Malnutrition in bariatric-treated patients after surgery is also documented; for example, the restrictive diet can lead to deficits of key nutrients such as B_12_, folic acid, calcium, and iron, all requiring surveillance and replacement [26,27,28]. The financial burden of specialized medical foods and formulas is substantial and often not fully covered by insurance, further complicating long-term adherence [27]. Sapropterin dihydrochloride, a synthetic form of the natural *PAH* cofactor BH_4_, can increase residual *PAH* activity in some patients, thereby lowering blood *Phe* levels (Table 1). However, only approximately 20–30% of PKU patients respond to BH_4_ therapy, primarily those with mild or moderate genotypes retaining some *PAH* function [29,30,31]. Patients with classic PKU or null mutations typically do not benefit (Table 2). Even among responders, sapropterin rarely allows complete dietary liberalization, and most patients must continue nutritional restrictions. The long-term safety and efficacy of BH_4_ therapy remain under investigation, and the high cost may limit accessibility. As an alternative, pegvaliase is a PEGylated recombinant phenylalanine ammonia lyase enzyme that degrades *Phe* independently of *PAH* (Table 1) [32]. It is a prescription medicine approved for adults with uncontrolled PKU. Despite pegvaliase’s ability to drive striking drops in blood *Phe*, it is also notorious for causing high rates of adverse events, some of which are potentially serious (such as injection site reactions and arthralgia) or truly severe dermatologic ones. It even carries the risk of anaphylaxis [33]. Given these side effects, close monitoring and immunomodulatory premedication are required. However, its use has been marred by low patient acceptance and adherence due to cumbersome administration and side effect profile. Pegvaliase is not indicated for children, but the relevance of this treatment might still be less given that clinical trials now exist in 12–17-year-olds [32,34,35]. It highlights the urgent requirement for disease-modifying therapies addressing the primary genetic defect that have the potential to provide lasting metabolic correction.

## 3. Gene Therapy Approaches for PKU

### 3.1. rAAV-Mediated Gene Therapy

Of the different approaches investigated in the field of gene therapy for monogenic liver disorders, including PKU, rAAV-based delivery of therapeutic genes has proven to be one of the most well-established (Figure 1, Table 1). Herein, we report that non-pathogenic and replication-incompetent adenoviruses encoding dynein-based motors can provide long-term transgene expression, most notably in quiescent cells such as hepatocytes that can re-enter the cell division cycle during certain physiological or pathological conditions. In preclinical models, lentiviral and adeno-associated virus (AAV) gene transfer to the liver is long-lasting. In contrast, other AAV vectors, such as *AAV8* or Rhesus isolate 10, readily transduce hepatic tissue and are capable of inducing sustained *PAH* expression after a single intravenous administration [36,37,38,39]. In a PKU mouse model (*Pah^enu2^*), liver-directed delivery of *AAV8* carrying a codon-optimized human *PAH* cDNA restored blood Phe levels to near-normal levels for over one year post-injection [40,41,42,43,44]. Introducing *FGF21* specifically within muscle using AAV vectors gave comparable results, but systemic metabolic correction is overall more efficient following hepatic delivery [13]. As a result, rAAV vectors represent the workhorse for gene addition therapies and can provide long-lasting transgene expression in a benign safety system. A practical problem of AAV therapy is that host immune responses against the capsid or transgene product may diminish efficacy or block re-dosing. Current research holds promise to increase their usage in monogenic and complex diseases [45].

### 3.2. Base Editing Strategies Using CRISPR-Cas9

Base editing is a new generation gene-editing platform that can correct mutations causing diseases to the level of single-nucleotide precision. In contrast, base editors—such as ABEs and the cytosine base editor (CBE)—differ from conventional editing in that they mediate a direct chemical conversion of one base pair to another by deaminase enzymes rather than double-strand break repair. In addition, its edits are limited to a narrow 3–5 nt region in the protospacer adjacent motif. Specificity is generally enhanced by narrow windows (1–2 nt) [46,47]. For example, *ABE8.8* offers several advantages in targeting accuracy and reduction of off-target activity across a wide range of models compared to the earlier *Cas9* enzyme [48]. LNP-encapsulated *ABE8.8* mRNA efficiently targeted and rescued the pathogenic allele in hepatocytes [49]. Remarkably, *Phe* concentrations returned to normal in as few as 48 h and remained approximately normal for more than 24 weeks, suggesting a durable therapeutic effect of base editing. Critically, this strategy was proposed to translate effectively to and was tested in both mice and non-human primates without significant evidence of hepatotoxicity or off-target mutagenesis. Deaminase enzymes greatly help in transition mutations; hence, this rundown is most appropriate to CBEs and ABEs. Other base editors that target transversion mutations (conversion between purines and pyrimidines) possess different molecular workings than CBEs, but those will not be covered in this review.

### 3.3. LNP Delivery Systems

LNPs is the widespread use as non-viral delivery systems, delivering mRNA or gene editing molecules to the several organs (Figure 1, Table 1). Many groups applied mRNA vaccines against COVID-19 to validate [50]. The LNPs are non-integrating into the genome, have a lower immunogenic risk compared with viral vectors, and can be administered repeatedly [51]. Concerning base editing, *ABE8* reduced *Phe* level in serum on the second day in mice [48]. Further, this approach improved on-target editing efficiency (by up to 61%) and significantly decreased bystander and off-target effect. This might be useful in pediatric patents.

## 4. Animal Models of PKU

Such animal models are extremely valuable for the evaluation of methods aimed at validating new therapeutic strategies, studying disease pathogenesis, and assessing long-term treatment. To date, different transgenic models of PAPPA2 have been generated in mouse, rat, and pig by germline mutagenesis or knock-in (KI) of clinically relevant *PAH* mutants. Table 3 provides a comparative summary of representative PKU models and their genetic modifications, phenotypes, and experimental purposes. A tabular listing of the major characteristics is beneficial in choosing a model that best matches research aims and therapeutic modalities.

### 4.1. Germline Mutagenesis for PKU Models

To develop the germline mutagenesis model of PKU, we developed the in-breadline BTBR mouse treatment and PKU mimic model using N-ethyl-N-nitrosourea (ENU), which enhances chemical mutagenesis. The ENU produced random point mutations in the PAH gene, accumulated Phe in the blood, followed by selective screening. *Pah^enu1^* is identified on a screen that detects a new allele in BTBR [52]. This mimic mutation in the PKU has only mild elevated serum *Phe* and mild neurological defects, and this mouse is not ideal for modeling the entire range of disease phenotypes found in the PKU. This locus-specific follow-up corresponds to a subsequent non-complementary screen and lead to the identification of *Pah^enu2^* and *Pah^enu3^*. These two impact alleles show a significant phenotype with a more than 20-fold of *Phe* concentration in serum and less than 5% liver *PAH* activity. These phenotypes demonstrated systemic and fine motor abnormalities and behavioral disorders. In particular, the three allele mutations have levels of cross-reactive *PAH* protein expression with different *Pah* mRNAs, and their molecular deficiencies do not necessarily define the severity of the allele phenotype (Table 3). In conclusion, many germline mutagenesis models have emerged in recent years to provide a fixed conceptual basis for dietary intervention and neurodevelopmental endpoints that are likely relevant, from current PKU treatment to gene- and cell-based treatments.

### 4.2. CRISPR/Cas9 Knockout (KO) Models

To eliminate residual expression of inactive *PAH* proteins and the dominant-negative interference observed in *Pah^enu2^* mice, CRISPR/*Cas9* gene editing was used to generate completely destructive *Pah-KO* mice (Table 3) [53]. An early stop codon (*GAG→TAG*) was introduced into codon 7 in exon 1 of the *Pah* gene, resulting in a complete loss of *PAH* expression at both the transcript and protein levels. Unlike *Pah^enu2^* mice, which produce a catalytically inactive but immunologically cross-reacting *PAH* protein, the *Pah-KO* model removes background signals and allows clear quantification of *PAH* for exogenous treatments. This improves the interpretability of efficacy studies. The construct was introduced into C57BL/6J embryos by means of microinjection of Cas9 mRNA, guide RNA, and a donor oligonucleotide. Of the 64 viable pups generated, two founders harbored the targeted mutation; one founder (#5349) was selected for colony expansion based on consistent hyperphenylalaninemia, reduced Tyr levels, and normal hepatic enzyme values. Homozygous KOs showed a wide range of phenotypic features characteristic of classical PKU, including high blood and brain Phe levels, neurotransmitter deficiencies, hypomyelination of the corpus callosum, growth retardation, and reduced body weight gain. Hypo-pigmented fur appearance was caused by hair follicle melanin deficits; bilateral cataracts developed several months after birth, possibly due to long-term exposure of developing lens tissue to high concentrations of *Phe* in the blood, and impaired nest-building behavior. The *Pah-KO* model is an excellent tool for preclinical evaluation of *PAH*-restoring therapies, including AAV or LNP-based gene transfer, mRNA delivery, and genome editing. The null background of the latter allows accurate definition of expression and functional rescue without pleiotropism with endogenous mutant *PAH*. In addition, the model faithfully recapitulates many of the molecular, metabolic, neuropathological, and behavioral phenotypes of severe murine PKU, enabling robust models to investigate pathogenesis in vivo or evaluate potential therapies.

### 4.3. CRISPR-Based Pah-R261Q KI Mouse

A CRISPR/*Cas9*-mediated genome editing approach was applied to create a *Pah-R261Q* KI mouse by introducing a mutation (*c.782G>A; p.R261Q*) commonly observed in the clinic into exon 7 of the murine *Pah* gene (Table 3) [54]. This precise integration results in the recapitulation of an ordinary human PKU-associated genotype at its natural genomic location. In this respect, these models contrast with ENU-induced mutagenesis models, which tend to produce broader (often null-like) phenotypes and do not allow for fine allelic control. The resultant mice had ~15% of the regular hepatic *PAH* activity, in good agreement with the residual enzymatic function in *R261Q*-homozygous patients. Yet these mice showed only mild hyperphenylalaninemia at baseline, perhaps because expression of hepatic *PAH* has species-specific differences. Of note, this model also gave us a new view that there is complex and unique molecular pathology, not limited to a simple enzyme deficiency. The PAH protein of the p.*R261Q mutant* is very conformationally unstable, forming of amyloid-like cytosolic foci. These aggregates colocalize with autophagosome with p62 and *LC3*, and have a high tendency to aggregate into β-sheet structures. In contrast to the smaller aggregates found in ENU-derived *Pah-V106A* models, those observed in *Pah-R261Q* models are numerous as well as extranuclear and impart oxidative stress on non-causal regions, all without Phe elevation, supporting a toxic gain-of-function pathology. These aggregates also align with increased antioxidant capacity, changes to lipid metabolism, and upregulation of *Dnajc12*, a lung *PAH*-specific co-chaperone. This finding implicates protein misfolding in metabolic dysfunction. Thus, the *Pah-R261Q* KI model offers a new, well-defined genotype-based preclinical platform. Its well-defined genetic makeup allows for humanized-type strategies, e.g., allele-specific gene editing or pharmacological chaperones, that are not translatable in null or ENU-based models. Furthermore, it can be used in the study even if we do not perturb metabolic status and assess how adiposity relates to redox imbalance in PKU comorbidities. It thus provides a key link between enzyme deficiency—on which current therapeutic strategies hinge—and the expression of systemic disease. It may help guide patient stratification efforts toward mutation-specific aggregation propensity.

### 4.4. Prime Editing in Humanized PKU Models

The *c.1222C>T (p.Arg408Trp)* mutation in the *PAH* gene represents the most prevalent pathogenic allele among individuals with PKU, particularly in European populations. In light of the limited efficacy of existing therapies in individuals carrying this severe mutation, recent efforts have centered on prime editing—a genome editing technology capable of precise nucleotide substitutions without inducing double-strand breaks. In this study, researchers developed both an in vitro hepatocyte model and a humanized PKU mouse model harboring the *c.1222C>T* variant through targeted replacement of murine genomic loci with corresponding human PAH sequences (Table 3) [14]. The homozygous *R408W* mice exhibited elevated blood *Phe* levels, hypopigmentation, and growth retardation, which is consistent with the classical presentation of PKU. Utilizing a dual *AAV8* delivery system encoding a split prime editor and an optimized pegRNA/nicking gRNA pair (*P56/N19*), the study attained up to 52% editing in whole-liver tissue and complete normalization of blood *Phe* levels, even in compound-heterozygous mice. Here is what the authors of this work say about these prime editing strategies for in vivo correction of a human *PAH* variant with clinical significance: “This approach was vastly more effective than prior nuclease- or base-editing-based attempts, both in terms of efficiency and the capacity to restore the phenotype.” Furthermore, stringent off-target analysis using ONE-seq and deep sequencing strategies did not detect any substantial off-target effects. Although the efficacy results were promising, challenges remain with AAV vectors in particular. These barriers have implications for durable expression of the transgene, immune responses, and potential for re-dosing. On the other hand, LNP-mediated delivery provides an opportunity to overcome these obstacles. Still, at present, its ability to deliver prime editing in vivo is limited by technical challenges related to payload size and complexity. However, the humanized model and algorithm to improve prime editing efficiencies for this disease presented here offer a powerful platform for the preclinical assessment of prime editing therapies not only for PKU but also for other rare monogenic liver disorders.

### 4.5. CRISPR/Cas9-Mediated Pah Exon 1 Deletion Model

CRISPR/Cas9 genome editing was used to develop a novel murine model (Table 3) [55]. The KI mouse carries an excisable deletion of exon 1 of the *Pah* (ΔExon1) model. Guide RNAs that target the 5′ untranslated region and intron 1 flanking exon 1 were tested in vitro and resulted in successfully targeted embryos, generating founder mice with a 258-bp deletion of exon 1. Homozygous ΔExon1 mice show the classical PKU phenotype with serum *Phe* concentrations over 2000 μM and severe brain accumulation of *Phe* (>800 nmol/g). Immunofluorescence and Western blot also revealed the complete lack of PAH protein in the liver. This class of compounds is distinct from previous models in which non-polymerized PAH monomers were still present. Not surprising given the depletion of 5-HT and 5-HIAA we previously observed in *Pah^enu2^* brain tissue. However, dopamine levels were unchanged, which is consistent with the usual PKU neuropathophysiology. In summary, this complete KO model appears to have distinct advantages for preclinical gene therapy studies. Of note, the absence of endogenous PAH protein in the sample rules out interfering signals in immunoassays, allowing accurate detection and quantification of transgene-derived PAH expression upon gene addition or editing. *Pah^ΔExon1^* mice treated with either dose of *AAV2/8-LSPmPAH* demonstrated a significant restoration of hepatic *PAH* activity that was dose-dependent and highly efficient, reducing blood *Phe* levels by 80–90% in heterozygotes to the same level as WT littermates, completely reversing the behavioral deficit when given at birth. They observed that these *Pah^enu3^* mice showed better therapeutic responses than the *Pah^enu2^* mice. In addition, heterozygous *Pah^+/Δexon1^* mice had substantially more residual liver *PAH* activity (~58% of wild-type) than *Pah^+/enu2^* (~29%). This result shows that the exon 1-deleted model is a potent tool for isolating the effects of the transgene. Overall, the *Pah^Δexon1/Δexon1^* model demonstrates a pure and accurate genetic background for gene/cell therapy development in PKU. Given advances in AAV-based gene therapy in current clinical practice, this model is likely to be a key component of preclinical optimization and translational causality.

### 4.6. Rat Models

Although many gene therapy protocols have sought to achieve correction of plasma *Phe* levels in PKU, full clinical benefit is contingent on repair of central nervous system (CNS) function, particularly in the prefrontal cortex. To test this hypothesis, Diamond and colleagues developed a rat model of early-treated PKU that reflects the mild *Phe* elevations (13–17 mg/dL) frequently observed in patients following a *Phe*-restricted diet or partial therapeutic correction (Table 3) [56]. Chronic, low-level hyperphenylalaninemia was induced in the rats through α-methylphenylalanine and supplemental *Phe* (either pre- and postnatally or postnatally alone) in this study. These animals exhibited selective deficits in delayed alternation—an appetitively motivated task that is dependent on prefrontal cortex function—even at Phe levels lower than those seen in classic untreated PKU. They showed reduced dopamine turnover, particularly within the medial prefrontal/anterior cingulate cortex. We suggest that these discoveries provide evidence for the notion that a relatively subtle rise in plasma *Phe* can positively induce enduring changes in both cortical monoamine metabolism and frontal executive function. A notable finding was that the reduction in homovanillic acid levels was highly correlated with the decrease in performance on behavioral tasks. This observation highlights the fact that the functionality of the prefrontal dopaminergic system is susceptible to minor biochemical alterations. Although this study did not attempt gene editing directly, it provides a good behavioral and neurochemical tool for testing the efficacy of genetic correction strategies. It would be beneficial for determining whether CNS-specific phenotypes, such as dopamine-dependent cognitive function, are restored after genetic intervention. Diamond et al.’s study establishes a necessary experimental groundwork for understanding therapeutic benefit beyond reduction in the biochemical abnormalities of PKU, which will be required if successful therapies are to achieve both metabolic and functional rescue [56].

### 4.7. Pig Models

To close the translational gap between murine studies and clinical application, a humanized porcine model of PKU was generated via transcription activator-like effector nucleases (TALENs) (Table 3) [57]. This model contains the robust human *R408W* mutation in the *PAH* gene, one of the most common and severe mutations worldwide. It includes five contiguous single-nucleotide polymorphisms, mimicking the human native haplotype. Somatic cell nuclear transfer using genetically modified pig fibroblasts produced homozygous *PAH^hR408W/hR408W^* piglets displaying classical PKU symptoms, including hyperphenylalaninemia and total loss of PAH enzyme activity; these animals responded to a *Phe*-restricted diet. Quantifiable levels of PAH protein were detected in the liver; however, no residual enzymatic function was found, as predicted by the known clinical phenotype of people who have this specific mutation. This model allows in vitro gene editing with TALENs and CRISPR/*Cas9* ribonucleoproteins for homology-directed repair at the humanized target locus. The inclusion of a fully human sequence block at the *R408W* locus allows for direct applications of editing tools designed for human therapy, enabling robust measurement of on-target efficacy and off-target specificity without species-specific adaptation. In addition, the physiological and structural similarities between human and pig hearts make it more favorable to test viral or non-viral delivery systems, immunogenicity, and therapeutic thresholds in a clinically relevant context. Here, we report the generation of a clinically relevant model of PKU by disrupting exon 3 within the endogenous *PAH* gene in SSA pigs, recapitulating both genotype and phenotype of an orthologue’s human disease allele into pigs; thus, representing the first large-animal platform suitable for somatic gene editing therapy development, optimization, and pre-clinical validation. The low correction threshold for phenotypic rescue in PKU gives this translational potential particular importance. While gene editing strategies, including base and prime editing, advance to clinical translation, this model holds promise for playing an indispensable role in safety, efficacy, and deliverability profiling of next-generation genomic therapies targeted at PKU and other monogenic liver diseases. Another study used a different pig model and made a *PAH*-deficient pig model using CRISPR/*Cas9*-mediated genome editing to target exon 6 of the porcine *PAH* gene. The strategy resulted in frame-shifting deletions that eliminated enzymatic activity and recapitulated the human PKU genotype and phenotype (Table 3) [58]. First, CRISPR was designed and validated in silico using a hybrid hamster–pig somatic cell line (A317), followed by the injection of *Cas9* mRNA and synthesized sgRNAs targeting introns flanking exon 6 into zygotes. One germline-typical skin cell probably created two fetuses that were transferred to surrogate sows and became two founders carrying biallelic deletions. One pig (116-1) harbored compound heterozygous deletions in both *PAH* exons 6 and 6–7, and the other (116-2) was identified as being heterozygous. Long-range PCR, droplet digital PCR, and whole-genome sequencing for genotyping finally revealed a faithful on-target modification profile in the absence of detectable off-targets, which supports the high fidelity of porcine zygotic editing with CRISPR/*Cas9*. Among these, the *PAH*-null pig (116-1) showed marked hyperphenylalaninemia (serum *Phe* > 4000 μM), small body size, and hypopigmentation characteristic of human classical PKU. In addition, brain MRI showed ventricular expansion and cortical gray matter loss in agreement with decreased neurogenesis, but there was no clear behavioral or neurological deterioration. Consistent with treatment practice, serum *Phe* levels fell well within the normal range when compared to clinical management of people living with PKU by simply reducing dietary intake of *Phe*. There are several advantages of this porcine model over traditional rodent models. Pigs also have similar anatomy and physiology to humans, including gyrencephalic brain structures like primates, extended periods of postnatal neuronal development until adulthood, and metabolic regulation with a propensity for metabolic perturbations, including insulin resistance. As the system similar models of human neuropathology. Therefore, this model shows potential for evaluating next-generation gene therapy approaches under a more clinically appropriate environment.

## 5. Clinical and Trial Medicine

### 5.1. SAR444836

SAR444836, an investigational AAV-based gene therapy discovered by Sanofi, demonstrated in a Phase 1/2 clinical trial to be safe and well-tolerated (clinicaltrials.gov ID: NCT05972629) (Table 4) [59]. It is administered by one-time intravenous infusion of the human *PAH* gene to adult-age patients with classic PKU. The primary endpoints comprise safety and change in plasma *Phe* concentration, with a reduction of dietary protein restrictions as an exploratory outcome. The treatment period is 96 weeks. Preliminary safety data to date have been positive, with transient elevations in liver transaminases observed as the sole adverse event. Long-term assessment of transgene expression and immunogenicity of AAV vectors may be a complication in ongoing follow-up [27,44].

### 5.2. NGGT002

NGGT002 is a gene therapy candidate undergoing Phase I/II clinical evaluation in the United States and China (Table 4). This includes sponsor-initiated trials in China (NCT06687733) and the U.S. (NCT06332807), alongside an investigator-initiated trial in China (NCT06061614) [60,61,62]. The therapy utilizes a recombinant *AAV8*-based delivery system and targets high-prevalence *PAH* mutations associated with PKU. NCT06687733 evaluates three dose cohorts in adults aged 18 to 55 years with classic PKU characterized by severe *PAH* deficiency and no residual enzyme activity. Early findings from this trial showed that five of six patients treated with a high-dose regimen achieved normalization of blood *Phe* levels within three weeks, with one patient maintaining normal levels for 40 weeks [46]. This study provides important proof-of-concept evidence for NGGT002’s efficacy [62]. NCT06061614 is an investigator-initiated, open-label, dose-escalation study in adults aged 18 and older with PKU and confirmed *PAH* mutations. Participants receive a single intravenous administration of NGGT002 and are followed for safety and efficacy for up to 5 years. This trial focuses on long-term safety, durability of gene expression, and immune response [61]. NCT06332807 is a Phase I/II, open-label, multicenter study in adults aged 18 to 55 years with classic PKU. It includes two dose cohorts with staggered dosing of initial subjects. Based on safety and efficacy data, dosing may escalate, or cohorts may be expanded. Participants receive a single intravenous infusion of NGGT002 and are monitored for up to 5 years. This trial provides dose-ranging data critical for determining optimal dosing strategies and expanding treatment applicability [60]. The ongoing Phase I/II clinical trials are designed to assess safety, tolerability, and efficacy. The study protocols include single intravenous administrations of NGGT002 with dose escalation after safety reviews of initial participants, and expansion cohorts planned at adequate dose levels. Subjects are monitored for plasma *Phe, PAH* activity, immune responses, vector shedding, and long-term safety outcomes. Further expansion into pediatric populations and broader genotypes is expected in future trial phases.

### 5.3. HMI-102

HMI-102 is an investigational gene therapy developed by homology medicines for the treatment of PKU in adults (NCT03952156) (Table 4) [63]. It is an investigational gene therapy developed by homology medicines, designed to deliver a functional copy of the human PAH gene to hepatocytes using an AAV vector, specifically *AAVHSC15*. Preclinical studies in murine models of PKU demonstrated that a single administration of HMI-102 resulted in a rapid and sustained reduction of blood *Phe*, normalization of *Tyr* levels, and restoration of brain neurotransmitter metabolites for up to one-year post-treatment. In the phase 1/2 pheNIX trial, HMI-102 was generally well tolerated. Some participants experienced transient elevations in liver transaminases (ALT/AST), which were managed with immunosuppressive therapy (tacrolimus and corticosteroids) and protocol amendments. Notably, several subjects exhibited clinically meaningful reductions in blood *Phe* levels [44]. However, in February 2022, the FDA hold due to elevated ALT/AST levels. Nevertheless, in August 2022, homology medicines suspended enrollment and terminated the study to prioritize the development of its gene-editing candidate, HMI-103 [63].

### 5.4. BMN 307

BMN 307 is an AAV-based investigational gene therapy developed by BioMarin Pharmaceutical, which is in a Phase 1/2 clinical trial (Table 4) (NCT04480567) [64]. The treatment consists of a one-time intravenous infusion of an AAV vector encoding the human *PAH* gene, intended to achieve long-term correction of *Phe* metabolism in adults with PKU. The study includes adult patients with classical PKU, and the primary outcomes studied are safety and reduction in plasma *Phe*. For the most part, data have suggested BMN 307 is generally well tolerated, with transient liver enzyme elevations representing the commonest adverse events. A long-term follow-up is underway to assess the persistence of transgene expression and clinical efficacy [46].

## 6. Discussion

Gene therapy for PKU is a milestone in the development of treatments for inherited metabolic disorders. Precision gene editing tools, together with tissue-specific delivery systems, have expanded therapeutic possibilities to durable benefits. While the early results are promising, several essential issues need to be resolved before gene therapy enters mainstream therapeutic practice. AAV-mediated gene transfer and CRISPR-based base editing are two examples of alternative therapeutic strategies that have proven to be remarkably effective in preclinical PKU animal models. AAV-mediated gene transfer has been shown to result in persistent transgene expression and long-term correction of *Phe* metabolism, up to more than 1 year in some instances. The human hepatocyte turnover is also much higher, and the sustainability of expression in humans, especially in developing pediatric livers, has not been tested [40,65,66,67,68,69]. The use of LNPs for base editing leads to practical, long-lasting therapeutic efficacy in murine models. However, no long-term data are reported in humans [46,70,71,72]. Gene therapy will not be successful if it is not safe. The ability of the AAV vector to elicit an immune response against the viral capsid or transgene product may induce immunity that precludes redosing and diminishes efficacy [37,65,73,74,75,76]. Base editors, on the other hand, do not create double-strand breaks; they still must be rigorously screened for off-target editing [77,78,79]. Because of their transient expression and non-integrating nature, LNPs have an improved safety profile (only mild elevations in liver enzymes reversible with discontinuation) [12,80,81,82,83]. Rigorous safety tracking and optimization of delivery strategies will be critical in clinical translation. Existing gene therapy candidates mainly focus on individual high-prevalence mutations like *P281L*. Nonetheless, more than 1000 *PAH* mutations have been identified, and notably, some patients are not candidates for all the current targeted therapies [84,85,86]. There is a need to develop mutation-agnostic therapies, such as universal gene addition or multiplexed base editing systems, to expand the scope of therapeutics. Furthermore, the majority of ongoing trials are restricted to adults. Because early intervention could prevent severe long-term neurological damage, expanding into pediatric populations is imperative. Some long-term goals of PKU gene therapy research will be to further adapt delivery mechanisms for redosing and tissue-specific targeting, as well as continue applying new prime editing technologies and expanding the reach of universal editing methods that can treat multiple variants at once. AAV-delivered prime editors efficiently correct pathogenic *PAH* variants in humanized mouse models and hepatocytes. In vivo studies on the *c.1222C>T* (*p.Arg408Trp*) mutation of *PAH* were performed using the prime editor [14]. Both studies showed high editing efficiencies and phenotypic correction. At the same time, the levels of off-target effects were low, which underscores the promise of prime editing for accurate, long-term correction of PKU-associated mutations. In addition, optimizing guide RNA design and reducing off-target effects can further improve editing specificity through the use of predictive, AI-driven tools [87]. Moreover, there could be a case for synergistic benefits from combination therapies that incorporate gene editing alongside the supplementation of metabolic cofactors or dietary modulation.

As promising as these studies are, there are many controversies and outstanding questions. First, the long-term transgene expression in pediatric livers is controversial because of the more rapid hepatocyte turnover in this age group. Secondly, the immunogenicity of AAVs still constrains the re-administration possibilities, raising concerns for long-term treatments. The off-target and bystander effects for base and prime editing have not been fully resolved in human systems, though preclinical data are encouraging. A common problem in current clinical trials is the overrepresentation of adult patients and the underrepresentation of pediatric patients, resulting in an insufficient body of evidence to address pediatric safety and effectiveness.

**Figure 2:**
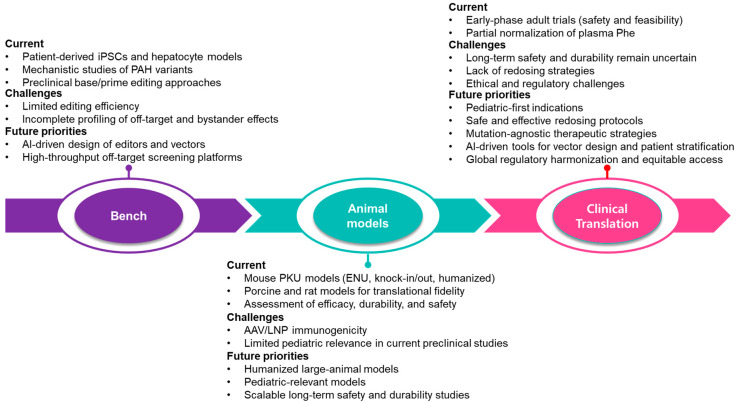
Future perspectives in PKU gene therapy: roadmap from experimental models to clinical application.

Furthermore, long-term safety, use in the pediatric population, and ethical and regulatory challenges continue to be central issues for clinical application of gene-editing technologies in rare metabolic diseases. Explicit direction from regulators and engagement with patients and families will be essential to ensure the responsible progression of clinical translation. A conceptual scheme is presented in Figure 2 to elucidate the translation pipeline of PKU gene therapy from bench to animal models and clinic, as well as the current developments, key bottlenecks, and future perspectives. These challenges will need to be tackled by the following approaches to achieve long-term clinical translation, such as improved delivery methods, strict long-term surveillance, and mutation-non-specific designs.

## 7. Conclusions

By providing a durable, potentially curative option to the lifelong burdens of diet and pharmacotherapeutics associated with the management of PKU, gene therapy has the potential to alter how this disorder is treated. Recently, the use of rAAV vectors and LNP-delivered base editors in the preclinical setting has paved the way for clinical translation. Partial normalization of plasma Phe has been reported in some patients, and the safety profiles in early-phase trials are encouraging. However, there are myriad challenges to durability and immunogenicity over the long term, heterotypic immunity, circulating variant strains that dominate the globe, and widespread use in children. This applies to all areas of gene therapy, from delivery platforms and editing tools to personalized medicine strategies. If confirmed by further research and clinical trials, gene therapy for PKU could eliminate the need for a diet in many cases for life and open up an era of truly individualized metabolic disease therapy. Looking forward, future research should prioritize the development of mutation-agnostic approaches that can broaden patient eligibility, the establishment of safe and effective redosing methods to overcome vector immunity, and the incorporation of AI-driven tools to optimize vector design and predict editing outcomes. Addressing these priorities will be essential for translating gene therapy in PKU from experimental promise to a durable and widely accessible clinical reality.

## Figures and Tables

**Table 1:** Comparison of Available and Investigational Therapies for PKU.

Therapy	Mechanism	Administration	Durability	Limitations
**Dietary Restriction**	Limit Phe intake	Daily diet	Lifelong	Poor adherence
**Sapropterin**	Cofactor supplementation	Oral tablet	Limited, mutation-specific	Only for mild cases
**Pegvaliase**	Enzyme substitution	Subcutaneous injection	Variable	Immunogenicity
**AAV Gene Therapy**	Gene addition (PAH)	IV infusion (1×)	Potentially long-term	Redosing issues
**Base Editing (LNP)**	Gene correction (PAH)	IV injection (1× or repeated)	Potentially permanent	Still under investigation

AAV, adeno-associated virus; LNP, lipid nanoparticle; Phe, phenylalanine; PAH, phenylalanine hydroxylase; IV, intravenous.

**Table 2:** The main PAH Mutations Associated with Poor Sapropterin (BH_4_) Responsiveness in PKU.

Mutation Name	Example Genotype(s)	Responsiveness	Notes/Description
***R408W***	*[R408W]; [R408W],* *[L348V]; [R408W]*	Very low/None	Most common nonresponsive mutation; catalytic domain
***IVS12+1G>A***	*[IVS12+1G>A]; [IVS12+1G>A]*	None	Splicing mutation; almost no residual enzyme activity
***P281L***	*[P281L]; [P281L]*	None	Catalytic domain; very low enzyme activity
**Null mutations** **(e.g., large deletions)**	*[Null]; [Null]*	None	No enzyme activity
***R158Q***	*[R158Q]; [R408W]*	None	Nonresponsive when combined with *R408W*
***L348V***	*[L348V]; [R408W]*	None	Nonresponsive when combined with *R408W*
***R261Q***	*[R261Q]; [R408W]*	None	Nonresponsive when combined with *R408W*
***Y414C***	*[Y414C];* [?]	Responsive	Known to be sapropterin-responsive
***L48S***	*[L48S];* [?]	Responsive	Known to be sapropterin-responsive

“None” indicates no significant reduction in blood Phe after sapropterin treatment. [?] indicates that the clinical phenotype associated with the corresponding PAH variant was not clearly reported or is currently undetermined.

**Table 3:** Comparative Analysis of PKU Models.

Model	Species	Key Mutations	Phenotype	Applications	References
Germline mutagenesis	Mouse	ENU-induced point mutation in *PAH*	Hyperphenylalaninemia, reversible with diet	Maternal PKU, gene therapy safety	[52]
Pah-KO (C57BL/6)	Mouse	Exon 7 stop codon	Classic PKU symptoms, hypomyelination	Preclinical drug testing	[53]
Pah-*R261Q*	Mouse	*R261Q* KI	Oxidative stress, protein aggregation	Mutation-specific biomarker discovery	[54]
*R408W*	Mouse	Humanized *PAH* exon 12 *(c.1222C>T; R408W)* KI	Elevated blood *Phe*, hypopigmentation, and reduced body weight	Model for testing gene editing strategies	[14]
ΔExon1	Mouse	CRISPR/*Cas9*-mediated deletion of exon 1 in PAH	Severe hyperphenylalaninemia, hypopigmentation, reduced serotonin and 5-HIAA in brain, undetectable *PAH* activity, partial perinatal	Preclinical evaluation of gene therapy; assessment of severe PKU pathophysiology and neurochemical imbalance	[55]
Early-treated PKU	Rat	Pharmacological *Phe*	Cognitive deficits, neurotransmitter loss	Neurodevelopmental studies	[56]
Humanized *R408W*	Pig	TALEN-mediated *R408W* KI	Human-like *PAH* dysfunction	Gene editing platform validation	[57]
CRISPR/*Cas9 PAH*-null pig	Pig (domestic sow × Yucatan minipig)	Biallelic exon 6 deletion in *PAH* via CRISPR/*Cas9* injection into zygotes	Severe hyperphenylalaninemia, hypopigmentation, growth retardation, high urinary phenylacetate, responsive to dietary Phe restriction, reduced cortical and cerebellar brain volume, no overt neurological deficits	Preclinical testing of PKU therapeutics, dietary treatment validation, neurocognitive and MRI endpoint assessment, maternal PKU studies	[58]

ENU, N-ethyl-N-nitrosourea (a chemical mutagen used for germline mutagenesis); KI, Knock-in; KO, Knockout; CRISPR, Clustered regularly interspaced short palindromic repeats (genome editing system); TALEN, Transcription activator-like effector nuclease; *PAH*, Phenylalanine hydroxylase; *Phe*, Phenylalanine; 5-HIAA, 5-Hydroxyindoleacetic acid (serotonin metabolite); MRI, Magnetic resonance imaging.

**Table 4:** Clinical trials of investigational gene therapies for PKU.

Therapy	Vector /Delivery	Clinical Stage	Patient Population	Administration	Outcomes	Limitations/ Current Status	Reference
**SAR444836** (Sanofi)	AAV-based (*PAH* transgene); IV single dose	Phase 1/2 (NCT05972629)	Adults with classic PKU	One-time IV infusion	Safe and well tolerated; transient liver enzyme elevations only	Long-term durability of transgene expression and AAV immunogenicity remain concerns; ongoing 96-week study	[59]
**NGGT002**	Recombinant AAV8; IV single dose	Phase 1/2 (NCT06687733, NCT06061614, NCT06332807)	Adults 18–55 yrs with classic PKU, severe *PAH* deficiency	Single IV infusion; dose-escalation cohorts	High-dose cohort: 5/6 patients normalized *Phe* within 3 weeks; durable response in some up to 40 weeks; designed for severe *PAH* deficiency	Need to confirm durability in long-term (5 years follow-up); safety monitoring for immune response and vector shedding; pediatric expansion planned	[60,61,62]
**HMI-102** (Homology Medicines)	AAVHSC15 vector; IV single dose	Phase 1/2 pheNIX trial (terminated) (NCT03952156)	Adults with PKU	Single IV administration	Preclinical: sustained *Phe* correction, restored *Tyr* and neurotransmitters; Early trial: clinically meaningful *Phe* reduction	FDA hold (2022) due to ALT/AST elevations; development halted (company shifted to HMI-103 gene editing)	[63]
**BMN 307** (BioMarin)	AAV-based; IV single dose	Phase 1/2 (NCT04480567)	Adults with classical PKU	One-time IV infusion	Generally well tolerated; transient liver enzyme increases; aimed at long-term *Phe* correction	Long-term persistence of effect still under evaluation; ongoing follow-up for efficacy and safety	[64]

AAV, adeno-associated virus; IV, intravenous; ALT, alanine aminotransferase; AST, aspartate aminotransferase; *Phe*, phenylalanine; *Tyr*, tyrosine; *PAH*, phenylalanine hydroxylase.

## Data Availability

The authors permit to share all the data.

## Abbreviations

The following abbreviations are used in this manuscript:

ABEs	adenine base editors
AAV	adeno-associated virus
AF	allele frequency
CNS	central nervous system
CBEs	cytosine base editors
enu	N-ethyl-N-nitrosourea
KI	Knock-in
KO	Knockout
LNPs	lipid nanoparticles
Phe	phenylalanine
PAH	phenylalanine hydroxylase
PKU	Phenylketonuria
rAAVs	recombinant adeno-associated viruses
TALENs	transcription activator-like effector nucleases
Tyr	tyrosine
